# Stress Elicits Contrasting Effects on Rac1-Cofilin Signaling in the Hippocampus and Amygdala

**DOI:** 10.3389/fnmol.2022.880382

**Published:** 2022-05-03

**Authors:** Mihika Bose, Mohammad Sarfaraz Nawaz, Rakhi Pal, Sumantra Chattarji

**Affiliations:** National Centre for Biological Sciences, Tata Institute of Fundamental Research, Bangalore, India

**Keywords:** chronic stress, synaptic plasticity, dendrite, spine, p-21 activated kinase

## Abstract

There is accumulating evidence for contrasting patterns of stress-induced morphological and physiological plasticity in glutamatergic synapses of the hippocampus and amygdala. The same chronic stress that leads to the formation of dendritic spines in the basolateral amygdala (BLA) of rats, leads to a loss of spines in the hippocampus. However, the molecular underpinnings of these divergent effects of stress on dendritic spines are not well understood. Since the activity of the Rho GTPase Rac1 and the actin-depolymerizing factor cofilin are known to play a pivotal role in spine morphogenesis, we investigated if alterations in this signaling pathway reflect the differential effects of stress on spine plasticity in the hippocampus and amygdala. A day after the end of chronic immobilization stress (2 h/day for 10 days), we found a reduction in the activity of Rac1, as well as its effector p21-activated kinase 1 (PAK1), in the rat hippocampus. These changes, in turn, decreased cofilin phosphorylation alongside a reduction in the levels of profilin isoforms. In striking contrast, the same chronic stress increased Rac1, PAK1 activity, cofilin phosphorylation, and profilin levels in the BLA, which is consistent with enhanced actin polymerization leading to spinogenesis in the BLA. In the hippocampus, on the other hand, the same stress caused the opposite changes, the functional consequences of which would be actin depolymerization leading to the elimination of spines. Together, these findings reveal a role for brain-region specific differences in the dysregulation of Rac1-to-cofilin signaling in the effects of repeated stress on two brain areas that are implicated in the emotional and cognitive symptoms of stress-related psychiatric disorders.

## Introduction

Stress-related psychiatric disorders are characterized by debilitating symptoms that include impaired cognitive function and heightened emotional problems. These contrasting manifestations at the behavioral level are accompanied by structural and functional aberrations in several brain regions including the hippocampus and amygdala (Bremner et al., 1997; Shin et al., 2005; Lorenzetti et al., 2009; Popoli et al., 2011). Consistent with these clinical findings, decades of research using a wide range of animal models have demonstrated how exposure to stress leads to divergent forms of morphological and physiological plasticity in neurons and their connections in the hippocampus and amygdala (Luine et al., 1994; Vyas et al., 2002; Mitra et al., 2005; Roozendaal et al., 2009; Chattarji et al., 2015). For instance, pioneering studies in various sub-regions of the rodent hippocampus reported dendritic shrinkage and reduction in spine numbers following chronic restraint stress (Watanabe et al., 1992). Subsequent analyses in the basolateral amygdala (BLA), by contrast, showed that chronic immobilization stress leads to the opposite effect—dendritic growth and spine formation (Vyas et al., 2002; Mitra et al., 2005). These divergent morphological effects are also accompanied by physiological alterations in synaptic plasticity—impaired long-term potentiation (LTP) in the hippocampus (Diamond and Rose, 1994; Kim and Diamond, 2002), but enhanced LTP in the BLA (Suvrathan et al., 2013). Further, consistent with these cellular changes, stress also impairs hippocampus-dependent spatial learning and memory (Luine et al., 1994; Popoli et al., 2011) but facilitates amygdala-dependent fear learning (Conrad et al., 1999; Bauer et al., 2002; Rau et al., 2005; Suvrathan et al., 2013). However, little is known about the molecular underpinning of these contrasting patterns of stress-induced changes at multiple levels of neural organization.

The present study is aimed at addressing this gap in knowledge by focusing on the opposite effects of stress on dendritic spines, the site of glutamatergic excitatory synaptic transmission. Dendritic spines are enriched in actin, a cytoskeletal protein that regulates spine shape and maintains spine stability (Cingolani and Goda, 2008; Hotulainen and Hoogenraad, 2010; Koleske, 2013). The actin-binding proteins cofilin and profilin are involved in actin depolymerization and actin polymerization respectively and play a central role in spine morphogenesis, and the addition and removal of synapses (Pontrello and Ethell, 2009; Hotulainen and Hoogenraad, 2010; Rust, 2015). The phosphorylation and inactivation of cofilin, in turn, are mediated by the Rho family of small guanosine triphosphatases (GTPases), primarily consisting of RhoA and Rac1 (Govek et al., 2005). Moreover, Rac1 is known to be a central regulator of actin cytoskeletal dynamics in dendritic spines thereby exerting control over the structural and functional plasticity of spines (Nakayama et al., 2000; Tashiro et al., 2000; Haditsch et al., 2009; Hedrick et al., 2016). Rac1 mediates phosphorylation of cofilin through its effector p21-activated kinase 1 (PAK1), leading to spine remodeling (Govek et al., 2005; Costa et al., 2020).

Although exposure to repeated stress causes spine removal in the hippocampus and addition in the BLA, whether stress causes any perturbations in Rac1-to-cofilin signaling in these two brain areas remains unexplored. For instance, would repeated exposure to stress affect GTPase activity and would these effects be different in the hippocampus vs. BLA? Further, would the same chronic stress elicit divergent effects on Rac1-cofilin signaling in the two areas? If so, would these stress-induced changes be consistent with the opposite directions of spine density changes reported in the two structures? Here we address these questions using a well-characterized model of chronic immobilization stress in rats (Vyas et al., 2002; Mitra et al., 2005; Rahman et al., 2016).

## Materials and Methods

### Animals

Eight-week-old male Sprague Dawley rats were pair housed in a standard 14 h light and 10 h dark schedule. Rats were housed under controlled humidity and temperature conditions with *ad libitum* access to food and water. All the experimentation procedures were approved by the Institutional Animal Ethics Committee, National Centre for Biological Sciences, Bangalore, India.

### Stress Protocol

Rats were subjected to chronic stress as per previously established protocols (Vyas et al., 2002; Mitra et al., 2005; Rahman et al., 2016). Briefly, chronic stress consisted of complete immobilization for 2 h per day for consecutive 10 days in plastic rodent immobilization bags without access to food and water. Prior to stress, rats were handled for three consecutive days and randomly divided into two groups—control and stress at the beginning of the experiment. Rats were sacrificed on the 11th day for further experiments.

### Body Weight

To calculate percentage gain in body weights, the net change in body weight of rats between the beginning and end points of the experiments was divided by the starting weight and multiplied by 100.

### Coronal Slice Preparation and Tissue Collection

Rats were anesthetized using CO_2_ on the 11th day, decapitated and their brains were rapidly dissected out and transferred to an oxygenated, ice-cold cutting solution composed of (in mM): 75 sucrose, 86 NaCl, 25 glucose, 2.5 KCl, 1.2 NaH_2_PO_4_, 25 NaHCO_3_, 7 MgCl_2_, 0.5 CaCl_2_; equilibrated with 95% O_2_and 5% CO_2_, pH 7.3, 305–310 mOsm. Coronal brain slices of 400 μm thickness containing hippocampus and amygdala were obtained in the cutting solution using Leica VT1200S vibratome (Leica, Germany). Dorsal hippocampus and basolateral amygdala were microdissected from the coronal slices, flash frozen, and stored at −80°C.

### Rac1 and RhoA Activation Assay

The G-LISA Rac1 Activation Assay Biochem kit (Cytoskeleton Biochem kit; Denver, USA; catalog no. BK128) and RhoA Activation Assay Biochem kit (Cytoskeleton Biochem kit; Denver, USA; catalog no. BK124) were used to measure the activity of Rac1 and RhoA respectively as per manufacturer’s protocol. The kits determine Rac1 or RhoA activity based on the detection of active Rac1 or active RhoA protein bound to GTP. The G-LISA assay uses a 96-well plate coated with either Rac1-GTP binding protein or Rho-GTP binding protein. Active, GTP-bound Rac1 or RhoA in tissue lysate bound to the wells while inactive GDP-bound Rac1 or RhoA were removed during washing steps. The bound active Rac1 or RhoA were detected after incubation with specific Rac1 or RhoA primary antibody respectively followed by HRP-conjugated secondary antibody. The absorbance was measured at 490 nm using a microplate reader (Tecan Spark, Switzerland).

### Synaptoneurosome Preparation

Synaptoneurosomes were prepared from the dorsal hippocampus or basolateral amygdala by differential filtration as described previously with slight modification (Scheetz et al., 2000; Muddashetty et al., 2007). Briefly, microdissected tissue was homogenized at 4°C in 10 volumes of homogenization buffer [composed of (in mM): 118 NaCl, 4.7 KCl, 1.2 MgSO_4_, 2.5 CaCl_2_, 1.53 KH_2_PO_4_, 212.7 glucose, 1 DTT and 20 Tris-HCL, pH 7.4], supplemented with 2× protease inhibitor cocktail (Sigma-Aldrich), 1× phosphatase inhibitor cocktail 2 and 3 (Sigma-Aldrich). The tissue homogenate was passed through three 100 μm nylon mesh filters (Merck Millipore; NY1H02500), followed by one 11 μm nylon net filter (Merck Millipore; NY1102500) and then centrifuged at 1,000× *g* for 15 min. The pellets containing synaptoneurosomeswere resuspended and lysed in RIPA lysis buffer containing 50 mM Tris-HCl (pH 7.4), 1% TritonX, 0.5% Na-deoxycholate, 0.1% SDS, 150 mM NaCl, 1 mM Na_3_VO_4_, 1 mM EDTA, 1 mM PMSF, 2× protease inhibitor cocktail (Sigma-Aldrich), 1× phosphatase inhibitor cocktail 2 and 3 (Sigma-Aldrich). The protein concentrations were estimated using BCA Protein Assay Kit (Pierce).

### Western Blotting

Twenty micrograms of protein from whole tissue lysate or synaptoneurosomes were loaded and separated in a precast gradient gel (NuPAGE 4%–12% Bis-Tris Protein Gels, Thermo Fisher). The resolved proteins were then transferred to a nitrocellulose membrane in a Bio-Rad transfer apparatus. After that, membranes were washed with 1× Tris-buffered saline (TBS). Next, membranes were blocked with 1:1 TBS: Odyssey Blocking Buffer (LI-COR Biosciences, Lincoln, NE, USA) containing 0.1% Tween 20 for 2 h at room temperature followed by overnight incubation at 4°C with primary antibodies (listed below). After subsequent washing with 1× TBST, the membranes were incubated with secondary antibodies (1:10,000 IRDye 800 CW goat anti-rabbit IgG; 1:10,000 IRDye 680 LT goat anti-mouse IgG; LI-COR Biosciences) for 1 h at room temperature. After incubation with respective secondary antibodies, the membranes were washed in 1× TBST. The immunoblots were then dried and digitally scanned using the Fc Odyssey Infrared Imaging System, (LI-COR Biosciences). Densitometric analysis was carried out with the help of Licor Image Studio Lite software.

### Primary Antibodies

The following primary antibodies were used in this study: mouse anti-Rac1 (ARC03; 1:500; Cytoskeleton), mouse anti-RhoA (ARH04; 1:500; Cytoskeleton), rabbit anti-PAK1 (2602S; 1:1,000; Cell Signaling Technology), rabbit anti-phospho-PAK1/PAK2 (2606S; Phospho-PAK1 (Ser^144^)/PAK2 (Ser^141^); 1:1,000; Cell Signaling Technology), rabbit anti-cofilin (5175S; 1:1,000; Cell Signaling Technology), rabbit anti-phospho-cofilin (3313S; Phospho-cofilin (Ser^3^); 1:1,000; Cell Signaling Technology), rabbit anti-profilin1 (3237S; 1:1,000; Cell Signaling Technology), rabbit anti-GAPDH (2118S; 1:5,000; Cell Signaling Technology), rabbit anti-profilin2 (ab174322; 1:1,000; Abcam).

### Statistical Analysis

Statistical analysis of all the data was performed using GraphPad Prism Software (GraphPad software Inc., USA, version 6). Significance was assessed by means of Student’s t-test (unpaired, two-tailed) since the sample distributions were normal in the two groups being compared. In the graphs, all the data has been represented as mean ± SEM. For all data analyzed, *p* < 0.05 was considered to be statistically significant.

## Results

### Contrasting Effects of Stress on Rac1-GTPase Activation in the Hippocampus and Amygdala

**Figure 1:**
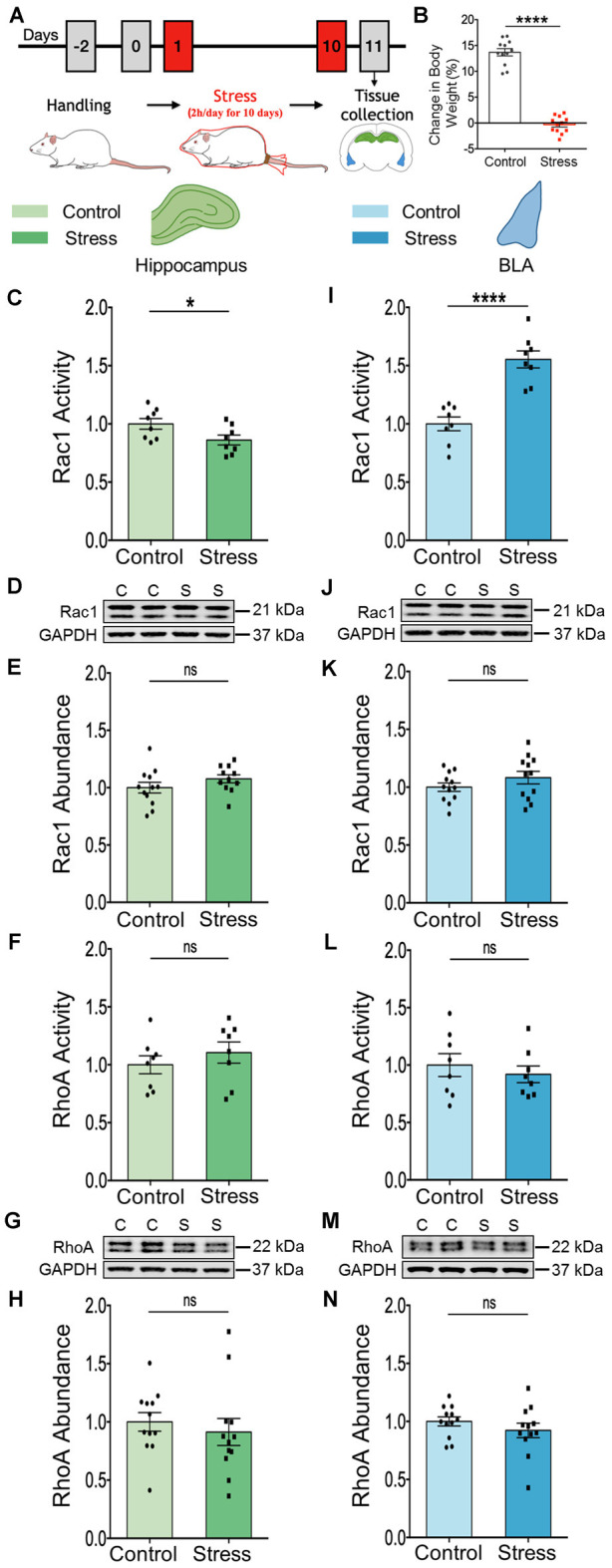
Chronic stress elicits contrasting effects on the activation profile of Rac1-GTPase in the hippocampus and amygdala. **(A)** Schematic representation of experimental protocol. Male rats were subjected to chronic immobilization stress for 2 h/day for 10 days. Animals were then sacrificed for tissue collection. **(B)** Rats subjected to chronic stress show a significant decrease in percentage gain in body weight compared to control rats. **(C)** Rac1 activity is significantly decreased in the hippocampus of the stressed rats compared to the controls. **(D,E)** No change in the total abundance of Rac1 protein in the hippocampus of the stressed rats has been observed as shown in the representative western blot **(D)** and the summary graph **(E)**. **(F–H)** No change in the activity of RhoA protein **(F)** or the abundance of the RhoA protein **(G,H)** has been observed in the hippocampus of the stressed rats compared to their control counterparts as shown in the representative blot **(G)** or the bar graph **(H)**. **(I)** Rac1 activity is significantly increased in the amygdala of the stressed rats compared to the controls. **(J,K)** No detectable difference in the total abundance of Rac1 protein has been observed in the amygdala of the stressed rats in comparison to control rats, as depicted in the representative blot **(J)** and the bar diagram **(K)**. **(L–N)** No change in the activity of RhoA protein **(L)** or the abundance of RhoA protein **(M,N)** in the amygdala of the stressed rats has been observed compared to control rats as depicted in the representative blot **(M)** and summary graph **(N)**. In the figure, C stands for control, S stands for stress and ns stands for non-significant. Data are represented as means ± SEM; data normalized to control animals except **(B)**; *N* = 8–12 rats/group; *p < 0.05, ****p < 0.0001.

First, we verified the efficacy of the chronic immobilization stress (Stress, 2 h/day for 10 days) paradigm (Figure 1A) by measuring the relative gain in body weight of rats. Relative to unstressed control rats, stressed rats underwent a significant reduction in the percentage weight gained 1 day after the end of stress (Figure 1B; Control: 13.69 ± 0.7%; Stress: −0.32 ± 0.47%; *N* = 12 rats/group; ^****^p < 0.0001). Next, we tested if changes in activation patterns of the Rho family of small GTPase may reflect the differential effects of stress on spine plasticity in the hippocampus and amygdala. To this end, we first measured the activity and abundance of Rac1 and RhoA GTPases in the whole tissue lysate obtained from the dorsal hippocampus and basolateral amygdala (BLA) from control and stressed rats. We performed ELISA based GTPase activation assay and immunoblotting was carried out to measure protein abundance. In the hippocampus, we observed a significant reduction of Rac1 GTPase activity in tissue collected from stressed compared to control rats (Figure 1C; Control: 1.00 ± 0.05; Stress: 0.86 ± 0.04; data normalized to control animals; *N* = 8 rats/group; *p < 0.05), but not in the total abundance of Rac1 protein (Figures 1D,E; Control: 1.00 ± 0.05; *N* = 12 rats; Stress: 1.08 ± 0.04; data normalized to control animals; *N* = 11 rats). However, there was no significant difference in the activity (Figure 1F; Control: 1.00 ± 0.08; Stress: 1.1 ± 0.09; data normalized to control animals; *N* = 8 rats/group) or abundance of RhoA protein (Figures 1G,H; Control: 1.00 ± 0.08; Stress: 0.91 ± 0.12; data normalized to control animals; *N* = 12 rats/group) in the hippocampus of stressed rats relative to controls. In contrast, the same chronic stress led to a significant increase in Rac1 GTPase activity in the BLA of the stressed animals relative to their control counterparts (Figure 1I; Control: 1.00 ± 0.06; Stress: 1.55 ± 0.07; data normalized to control animals; *N* = 8 rats/group; ^****^p < 0.0001) with no difference in total abundance of Rac1 protein (Figures 1J,K; Control: 1.00 ± 0.04; Stress: 1.08 ± 0.05; data normalized to control animals; *N* = 12 rats/group). Also, there was no change in the activity (Figure 1L; Control: 1.00 ± 0.10; Stress: 0.92 ± 0.07; data normalized to control animals; *N* = 8 rats/group) or abundance of RhoA protein (Figures 1M,N; Control: 1.00 ± 0.04; Stress: 0.92 ± 0.06; data normalized to control animals; *N* = 12 rats/group) in the BLA of stressed rats compared to controls. Taken together, these data indicate chronic stress modulates Rac1 GTPase activity in opposite directions in the hippocampus and BLA, without affecting RhoA activity.

### Contrasting Effects of Stress on PAK1 Activity in the Hippocampus and Amygdala

The PAK1 protein is a critical effector that links Rac1 GTPase activity to cytoskeleton remodeling (Zhao and Manser, 2012; Rane and Minden, 2014). This led us to investigate whether stress can differentially dysregulate PAK1 activity in the hippocampus and amygdala. First, we assessed PAK1 activity by examining its phosphorylation status, as well as abundance of PAK1 in the whole tissue lysate from both brain areas. There was a significant decrease in PAK1 activity in the hippocampus (\hyperref[s9]**Supplementary Figures 1A,B**; Control: 1.00 ± 0.07; Stress: 0.70 ± 0.05; data normalized to control animals; *N* = 8 rats/group; **p < 0.01 and \hyperref[s9]**Supplementary Figure 1E**; Control: 1.00 ± 0.05; Stress: 0.72 ± 0.06; data normalized to control animals; *N* = 8 rats/group; **p < 0.01), without any change in the total abundance of PAK1 protein (\hyperref[s9]**Supplementary Figures 1C,D**; Control: 1.00 ± 0.05; Stress: 0.99 ± 0.06; data normalized to control animals; *N* = 8 rats/group). In the BLA, however, there was no change in activity of PAK1 (\hyperref[s9]**Supplementary Figures 1F,G**; Control: 1.00 ± 0.11; Stress: 0.94 ± 0.11; data normalized to control animals; *N* = 8 rats/group and \hyperref[s9]**Supplementary Figure 1J**; Control: 1.00 ± 0.09; *N* = 8 rats; Stress: 0.99 ± 0.07; data normalized to control animals; *N* = 7 rats) or its abundance (\hyperref[s9]**Supplementary Figures 1H,I**; Control: 1.00 ± 0.05; Stress: 0.86 ± 0.05; data normalized to control animals; *N* = 8 rats/group).

**Figure 2:**
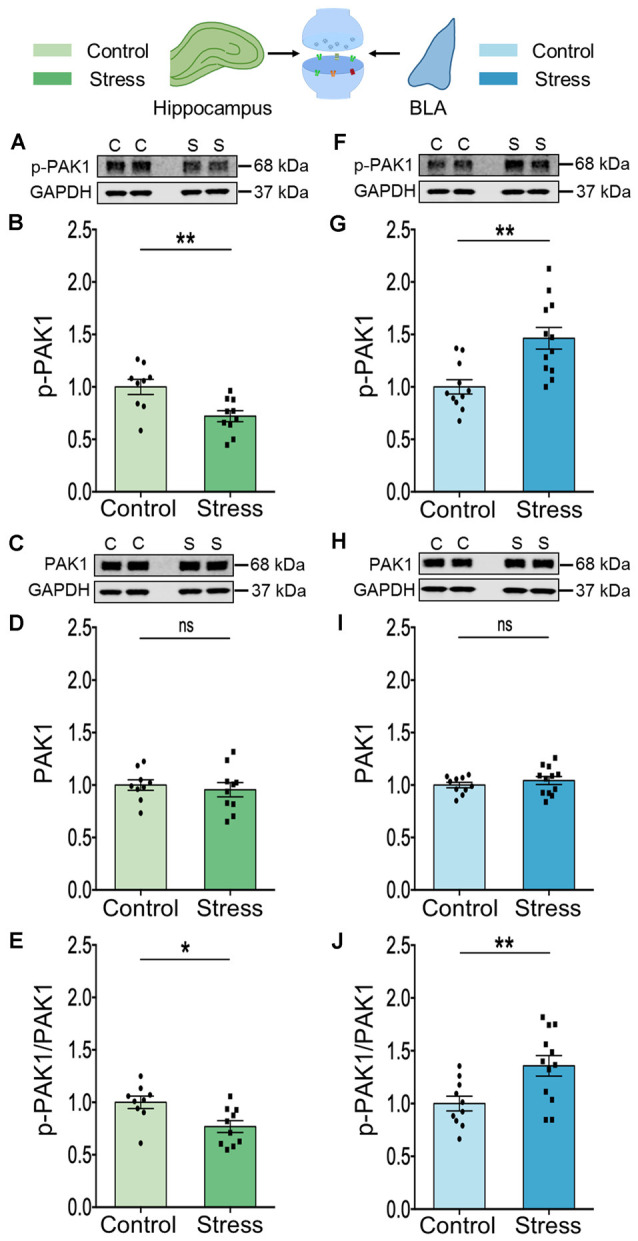
Chronic stress mediates contrasting effect on the activity of the effector molecule PAK1 in the synaptoneurosome fraction isolated from the hippocampus and amygdala. **(A,B)** A significant decrease in the activity of PAK1 protein as represented by a decrease in its phosphorylation status has been observed in the hippocampus of the stressed rats compared to the controls as shown in the representative western blot **(A)** and the summary graph **(B)**. **(C,D)** No change has been observed in the abundance of the PAK1 protein in the hippocampus of stressed rats compared to controls as shown in the representative blot **(C)** and the summary graph **(D)**. **(E)** A significant decrease in the ratio of phospho-PAK1 to total-PAK1 protein has been observed in the hippocampus due to stress confirming a decrease in PAK1 activity. **(F,G)** A significant increase in the activity of PAK1 protein as represented by an increase in its phosphorylation status has been noticed in the amygdala of the stressed rats compared to the controls as shown in the representative western blot **(F)** and the summary data **(G)**. **(H,I)** No detectable difference in the abundance of the PAK1 protein has been viewed in the amygdala of stressed rats compared to controls as shown in the representative blot **(H)** and the summary graph **(I)**. **(J)** A significant increase in the ratio of phospho-PAK1 to total-PAK1 protein has been observed in the amygdala due to chronic stress confirming an increase in PAK1 activity. In the figure, C stands for control, S stands for stress and ns stands for non-significant. Data are represented as means ± SEM; data normalized to control animals; *N* = 9–12 rats/group; *p < 0.05, **p < 0.01. For datasets in Figures 2, 3, the same GAPDH as internal controls has been used for analysis since they are from the same blots. Finally, it may be noted that the blots of p-PAK depicted in panels **(A)** and **(F)** have low signal-to-noise ratio, which gives the impression of a higher background resulting in a smear-like appearance. However, comparisons with Cell Signaling Technology (CST) datasheets for the respective antibodies, as well as previous articles where similar blots for phosphoproteins have been presented (Pyronneau et al., 2017; Brown et al., 2021), suggest that this is caused by a low signal-to-noise ratio.

Next, to gain a better understanding of stress-induced changes at the synaptic level for which analyses of whole tissue lysates are not optimal, we switched to measurements in synaptoneurosomes. To this end, we isolated synaptoneurosomes from both brain areas to quantify the activity and abundance of PAK1 protein by immunoblotting. In the hippocampus of stressed animals, we found a significant decrease in PAK1 phosphorylation, at Ser^144^ (Figures 2A,B; Control: 1.00 ± 0.07; *N* = 9 rats; Stress: 0.72 ± 0.05; data normalized to control animals; *N* = 10 rats; **p < 0.01 and Figure 2E; Control: 1.00 ± 0.06; *N* = 9 rats; Stress: 0.77 ± 0.06; data normalized to control animals; *N* = 10 rats; *p < 0.05). This is a primary phosphosite undergoing autophosphorylation upon PAK1 activation and regulates the enzymatic activity of PAK1 (Mayhew et al., 2007). Stress did not change total abundance of hippocampal PAK1 protein (Figures 2C,D; Control: 1.00 ± 0.05; *N* = 9 rats; Stress: 0.96 ± 0.07; data normalized to control animals; *N* = 10 rats). Notably, a significant increase in PAK1 phosphorylation at Ser^144^ was seen in the BLA of stressed rats (Figures 2F,G; Control: 1.00 ± 0.07; *N* = 11 rats; Stress: 1.46 ± 0.10; data normalized to control animals; *N* = 12 rats; **p < 0.01 and Figure 2J; Control: 1.00 ± 0.07; *N* = 10 rats; Stress: 1.36 ± 0.10; data normalized to control animals; *N* = 12 rats/group; **p < 0.01) without any detectable change in the total abundance of the protein (Figures 2H,I; Control: 1.00 ± 0.03; *N* = 10 rats; Stress: 1.04 ± 0.04; data normalized to control animals; *N* = 12 rats). However, PAK2 phosphorylation at Ser^141^ was not affected by stress in hippocampal (\hyperref[s9]**Supplementary Figures 2C,D**; Control: 1.00 ± 0.09; *N* = 9 rats; Stress: 0.89 ± 0.06; data normalized to control animals; *N* = 10 rats) and BLA (\hyperref[s9]**Supplementary Figures 2G,H**; Control: 1.00 ± 0.07; *N* = 11 rats; Stress: 1.10 ± 0.08; data normalized to control animals; *N* = 12 rats) synaptoneurosomes. A similar absence of stress effects was seen in analyses of PAK2 phosphorylation in whole tissue lysates obtained from both areas (\hyperref[s9]**Supplementary Figures 2A,B**; Control: 1.00 ± 0.06; Stress: 0.93 ± 0.08; data normalized to control animals; *N* = 8 rats/group and \hyperref[s9]**Supplementary Figures 2E,F**; Control: 1.00 ± 0.07; Stress: 0.97 ± 0.05; data normalized to control animals; *N* = 8 rats/group respectively). Together, these findings also demonstrate divergent effects of chronic stress on PAK1 activity, but not on PAK2, in the hippocampus and amygdala. Further, the decrease in PAK1 activity is consistent with reduced Rac1 activity in the hippocampus, while enhanced activity of both PAK1 and Rac1 was seen in the basolateral amygdala.

### Stress Also Leads to Divergent Effects on Cofilin Activity in the Two Brain Areas

**Figure 3:**
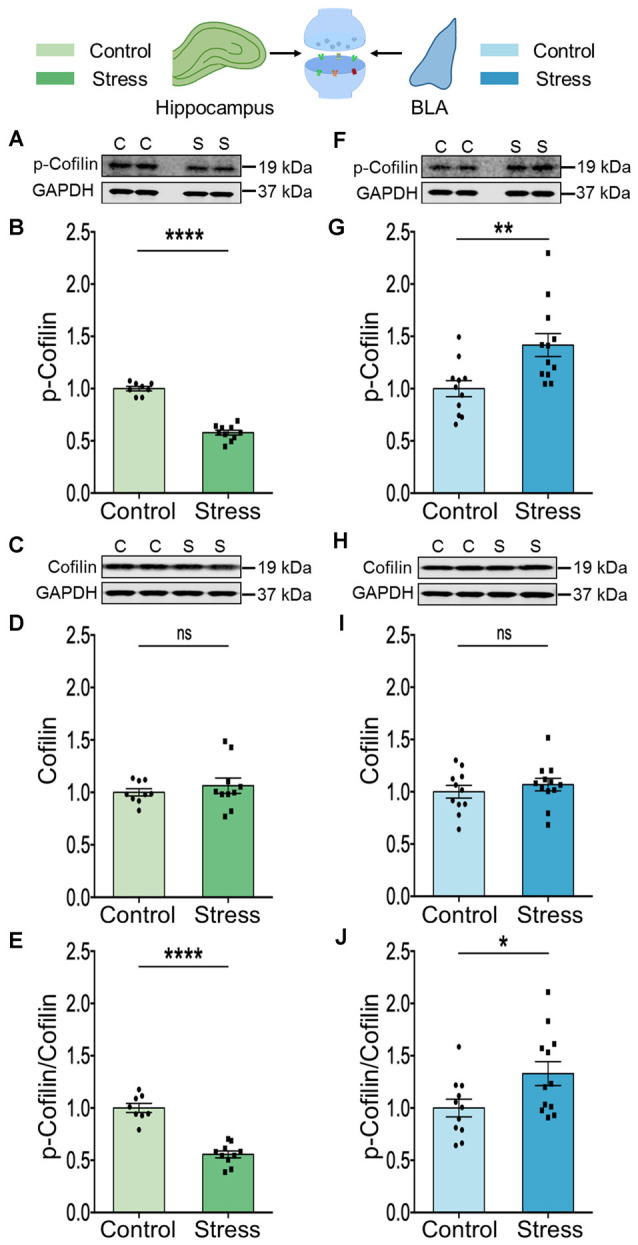
Chronic stress modulates cofilin activity in a differential manner in the hippocampus and amygdala. **(A,B)** A significant decrease in the phosphorylation of the cofilin protein has been observed in the hippocampus of the stressed rats compared to the controls suggesting an increase in the activity of cofilin, as shown in the representative western blot **(A)** and the summary graph **(B)**. **(C,D)** The abundance of the cofilin protein has been observed to undergo no change in the hippocampus of stressed rats compared to controls as shown in the representative blot **(C)** and the summary graph **(D)**. **(E)** A significant decrease in the ratio of phospho-cofilin to total-cofilin has been observed in the hippocampus due to stress confirming an increase in cofilin activity. **(F,G)** A significant increase in the phosphorylation of cofilin protein has been observed in the amygdala of the stressed rats compared to the controls suggesting a decrease in cofilin activity as shown in the representative western blot **(F)** and the summary data **(G)**. **(H,I)** No detectable difference in the abundance of the cofilin protein has been noticed in the amygdala of stressed rats compared to controls as shown in the representative blot **(H)** and the summary graph **(I)**. **(J)** A significant increase in the ratio of phospho-cofilin to total-cofilin has been observed in the amygdala due to stress confirming a decrease in cofilin activity. In the figure, C stands for control, S stands for stress and ns stands for non-significant. Data are represented as means ± SEM; data normalized to control animals; *N* = 8–12 rats/group; *p < 0.05, **p < 0.01, ****p < 0.0001. For datasets in Figures 2; 3, the same GAPDH as internal controls has been used for analysis since they are from the same blots. Finally, it may be noted that the blots of p-Cofilin depicted in panels 3A and 3F have a low signal to noise ratio, which gives the impression of a higher background resulting in a smear-like appearance. However, comparisons with Cell Signaling Technology (CST) datasheets for the respective antibodies, as well as previous articles where similar blots for phosphoproteins have been presented (Ouyang et al., 2021) suggest that this is caused by a low signal-to-noise ratio.

The results described thus far point to stress-induced changes in Rac1 and PAK1 (Figures 1, 2). Rac1 is known to exert its effects on spine architecture by modulating the activity of the actin-binding protein cofilin through PAK1. Moreover, cofilin plays a central role in regulating the structure and number of dendritic spines (Yang et al., 1998; Hotulainen et al., 2009; Pontrello and Ethell, 2009). Hence, we next compared the total levels of cofilin protein, as well as the phosphorylation status of cofilin at Ser^3^, in BLA, and hippocampal synaptoneurosomes. Stress led to a significant reduction in phosphorylation of cofilin at Ser^3^ in the hippocampus (Figures 3A,B; Control: 1.00 ± 0.02; *N* = 8 rats; Stress: 0.58 ± 0.02; data normalized to control animals; *N* = 10 rats; ^****^p < 0.0001 and Figure 3E; Control: 1.00 ± 0.04; *N* = 8 rats; Stress: 0.56 ± 0.03; data normalized to control animals; *N* = 10 rats; ^****^p < 0.0001) without any effect on total abundance of the protein (Figures 3C,D; Control: 1.00 ± 0.03; *N* = 9 rats; Stress: 1.06 ± 0.07; data normalized to control animals; *N* = 10 rats). In the BLA, by contrast, stress caused a significant increase in cofilin phosphorylation at Ser^3^ (Figures 3F,G; Control: 1.00 ± 0.08; *N* = 11 rats; Stress: 1.42 ± 0.11; data normalized to control animals; *N* = 12 rats; **p < 0.01 and Figure 3J; Control: 1.00 ± 0.08; *N* = 11 rats; Stress: 1.33 ± 0.11; data normalized to control animals; *N* = 12 rats; *p < 0.05) without any detectable difference in the total abundance of the protein (Figures 3H,I; Control: 1.00 ± 0.06; *N* = 11 rats; Stress: 1.07 ± 0.06; data normalized to control animals; *N* = 12 rats). Thus, stress-induced decrease in cofilin phosphorylation in the hippocampus, but increase in the BLA, is consistent with enhanced cofilin activity in the hippocampus, but the opposite effect in the BLA. Furthermore, the divergent effects of stress on Rac1-PAK1-cofilin signaling mirrors the loss and formation of spines in the hippocampus and BLA respectively.

### Stress Triggers Contrasting Patterns of Expression of Profilin Isoforms in the Two Brain Areas

**Figure 4:**
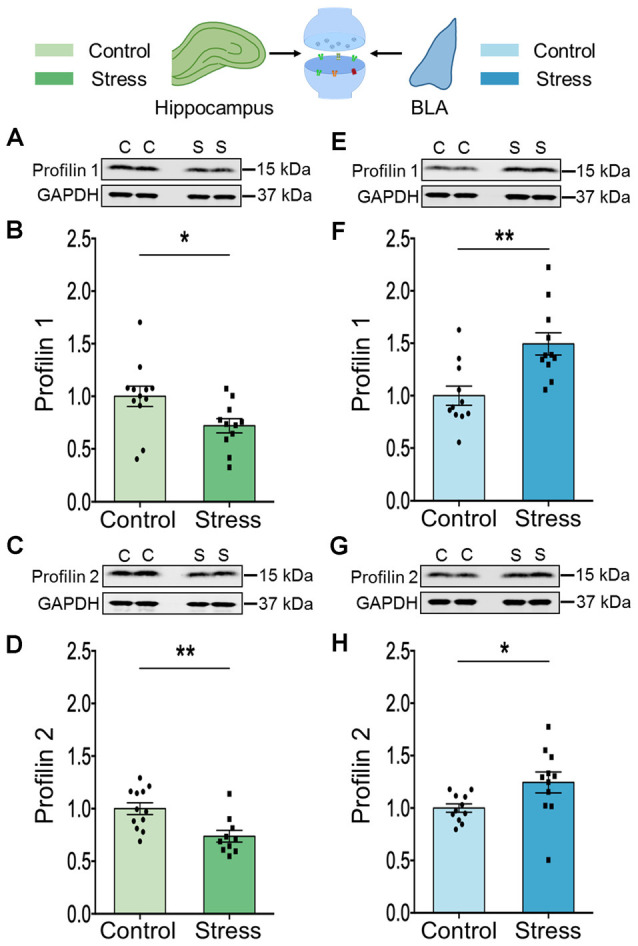
Chronic stress leads to contrasting effect on the expression profile of profilin isoforms in the hippocampus and amygdala. **(A–D)** A significant decrease in the expression of both the profilin isoforms, profilin 1 and profilin 2 has been observed in the synaptoneurosome fraction of the hippocampus of the stressed rats compared to controls as shown in the representative western blots **(A,C)** and the summary graphs **(B,D)** respectively. **(E–H)** A significant increase in the expression of both the profilin isoforms, profilin 1, and profilin 2 has been observed in the synaptoneurosome fraction of the amygdala of the stressed rats compared to its control counterparts as shown in the representative western blots **(E,G)**, and the summary graphs **(F)** and **(H)** respectively. In the figure, C stands for control and S stands for stress. Data are represented as means ± SEM; data normalized to control animals; *N* = 10–12 rats/group; *p < 0.05, **p < 0.01. Out of 11 data points in **(F)**, five data points have used the same internal control for analysis as in Figures 2G; 3G since they are from the same blots.

Similar to cofilin, profilins are actin-binding proteins that also regulate neuronal actin dynamics. Profilins are known to bind G-actin, enhance actin polymerization and play an important role in signal-dependent fine-tuning of spine architecture (Michaelsen et al., 2010). We focused on the two isoforms, profilin 1 and profilin 2 because they are reported to undergo stimulus-dependent accumulation in the spines of excitatory neurons (Ackermann and Matus, 2003; Neuhoff et al., 2005; Lamprecht et al., 2006). In the hippocampus, stress led to a reduction in the levels of both profilin 1 (Figures 4A,B; Control: 1.00 ± 0.10; *N* = 12 rats; Stress: 0.72 ± 0.07; data normalized to control animals; *N* = 11 rats; *p < 0.05) and profilin 2 (Figures 4C,D; Control: 1.00 ± 0.06; *N* = 12 rats; Stress: 0.74 ± 0.06; data normalized to control animals; *N* = 10 rats; **p < 0.01). However, in the BLA stress had the opposite effect on both isoforms (Figures 4E,F; Control: 1.00 ± 0.09; Stress: 1.5 ± 0.11; data normalized to control animals; *N* = 11 rats/group; **p < 0.01 and Figures 4G,H; Control: 1.00 ± 0.04; Stress: 1.24 ± 0.10; data normalized to control animals; *N* = 11 rats/group; *p < 0.05).

## Discussion

**Figure 5:**
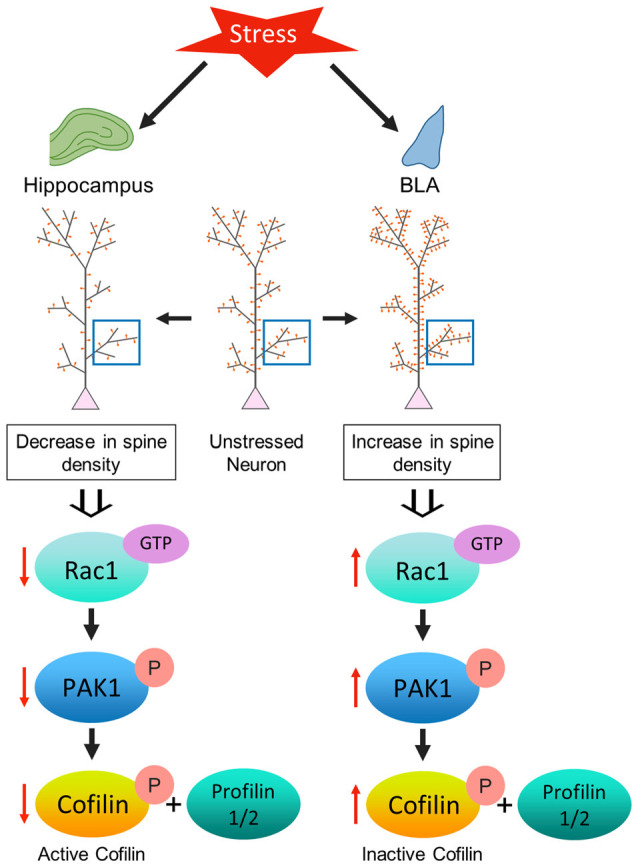
Model depicting the proposed mechanism underlying the contrasting effect of stress-induced structural plasticity in the hippocampus and amygdala. **(Left)** Schematic representation of the effect of stress on the hippocampus. Chronic stress leads to an aberrant decrease in Rac1 activity in the hippocampus which in turn leads to a decrease in PAK1 phosphorylation. A decrease in PAK1 activity ultimately brings about a decrease in cofilin phosphorylation. An increase in cofilin activity along with a simultaneous decrease in the levels of profilin isoforms may correlate with a decrease in spine density in the hippocampus due to stress. **(Right)** Schematic representation of the effect of stress on the amygdala. Chronic stress leads to an aberrant increase in Rac1 activity in the amygdala that effectuates an increase in PAK1 phosphorylation. An increase in PAK1 activity causes an increase in cofilin phosphorylation. A decrease in cofilin activity along with a simultaneous increase in the profilin isoforms may be linked with an increase in spine density in the amygdala due to stress.

The analysis presented here is one of the first attempts to characterize the impact of repeated stress on molecular signaling mechanisms underlying spine plasticity in two brain regions that play a pivotal role in regulating the stress response. We found that consistent with the divergent patterns of stress-induced structural plasticity in the hippocampus and amygdala, chronic stress also elicits differential changes in Rac1-PAK1-cofilin signaling and levels of profilin isoforms in these two structures (Figure 5). A day after the end of 10 days of chronic stress, we observed contrasting effects in the activation profile of Rac1—a reduction in the hippocampus, but an enhancement in the BLA. However, no detectable difference in RhoA activity was seen in either areas, suggesting that a dysregulation of Rac1 signaling, but not RhoA, is associated with chronic stress-induced structural plasticity. This is interesting in light of an earlier study that reported the involvement of Rac1 activation, but not RhoA, in antidepressant effects (Kato et al., 2018). Stress, in turn, has been implicated in precipitating depressive symptoms (Duman et al., 2019) and animal models of chronic stress have also been used to examine mechanisms of antidepressant action (Govindarajan et al., 2006; Pillai et al., 2012). Consistent with previous studies (Nakayama et al., 2000; Tashiro et al., 2000; Hedrick et al., 2016; Pyronneau et al., 2017), our results demonstrate the importance of Rac1 signaling in the structural plasticity of dendritic spines. RhoA signaling, on the other hand, has been shown to have a complex relationship with spine stability. For instance, RhoA has been implicated in the reduction of spine density (Tashiro et al., 2000; Govek et al., 2005). But, RhoA-ROCK signaling can also phosphorylate and inactivate cofilin, which could promote rather than reduce spine stability (Koleske, 2013). Since Rac1-GTPase mediates its effects by binding and activating PAK1 (Hayashi et al., 2002; Zhao and Manser, 2012; Rane and Minden, 2014), we also analyzed synaptoneurosomes; this revealed lower PAK1 activity in the hippocampus, but the opposite effect in the BLA. A downstream target of Rac1 is the actin-depolymerizing factor cofilin, which upon phosphorylation at its Ser^3^ residue, becomes inactive. As a result, it fails to bind and sever F-actin (Yang et al., 1998). Cofilin-mediated cytoskeletal remodeling plays a significant role in dendritic spine morphogenesis and alterations in spine density (Hotulainen et al., 2009; Mizuno, 2013; Ben Zablah et al., 2020). Following exposure to repeated stress, we found a significant increase in cofilin phosphorylation in the BLA, which is indicative of reduced cofilin activity. In striking contrast, the same stress led to a decrease in cofilin phosphorylation, suggesting its activation, in the hippocampus. High cofilin activity in the hippocampus, in turn, would result in a shift toward actin depolymerization that is consistent with a stress-induced reduction in hippocampal spine density reported in earlier studies (Watanabe et al., 1992; Vyas et al., 2002). Conversely, the opposite effect of the same chronic stress on BLA cofilin activity would cause a shift towards elevated actin polymerization, and thereby cause increased spine density. Together, these findings show how the differential dysregulation of Rac1-PAK1-cofilin signaling is consistent with the contrasting effects of stress on spine numbers in the hippocampus vs. the amygdala.

These findings are also consistent with cofilin being the convergence point of Rac1 signaling that ultimately modulates actin cytoskeleton dynamics. Similar to cofilin, the other actin-binding protein profilin also plays an important role in regulating actin dynamics and spine architecture (Michaelsen et al., 2010; Michaelsen-Preusse et al., 2016). Both profilin1 and profilin2 are required for synaptogenesis, while the latter is also important for spine stability and plasticity (Michaelsen et al., 2010). Here, we report that exposure to chronic stress decreases levels of profilin1 and profilin2 expression in the hippocampus, but increases their levels in the BLA. This decrease in cofilin activity, alongside higher profilin levels, would be consistent with enhanced actin polymerization leading to spinogenesis in the BLA. In the hippocampus, on the other hand, the same stress increased cofilin activity but decreased profilin levels, which would lead to actin depolymerization and loss of spines.

Our findings using a rodent model of stress add to a growing body of evidence for the central role played by Rac1-PAK1-cofilin signaling in modulating spine plasticity in both *in vitro* and *in vivo* rodent models. For example, in the developing neuron, Rac1 has been shown to mediate spine formation and activity-induced changes in spine size (Murakoshi et al., 2011; Koleske, 2013), as well as spine stability in adult neurons (Nakayama et al., 2000; Koleske, 2013). Increased or decreased Rac1 signaling has also been associated with elevated and reduced spine density in slice cultures (Tashiro et al., 2000; Tashiro and Yuste, 2004), in primary neuronal cultures (Pennucci et al., 2019), and in mice (Bongmba et al., 2011; Huang et al., 2011; Ohashi, 2015). Reduction in spine density has been seen following knockdown of profilin1 and profilin2 as well (Michaelsen et al., 2010; Michaelsen-Preusse et al., 2016). In light of the pivotal role of Rac1 in learning and memory, this small GTPase and its downstream molecules have also been associated with a range of brain disorders. For instance, in a rodent model of Alzheimer’s disease, spine density is reduced due to cofilin activation, which has also been seen in patient brain tissue (Zhao et al., 2006; Shankar et al., 2007). Also, in a mouse model of Fragile X Syndrome, elevated activation of Rac1-PAK1-cofilin signaling has been linked to enhanced dendritic spine density in the somatosensory cortex (Pyronneau et al., 2017). Of relevance to the findings reported here, in a recent study using a rat model of depression, PAK1 gene expression was downregulated in the hippocampus whereas the Rac1 gene was upregulated in the amygdala (Andrus et al., 2012). It should also be noted that our study is based on analyses of synaptoneurosomes, a method that is not amenable to differentiating between excitatory and inhibitory neurons. Further, inhibitory interneurons are aspiny or spine-sparse (Sala, 2002; Goldberg et al., 2003; Sancho and Bloodgood, 2018), and comprise of a relatively smaller proportion of the total population of neurons in the hippocampus and amygdala (Perumal and Sah, 2021). Thus, the divergent effect of stress on Rac1 reported here is more likely to reflect alterations in excitatory neurons.

The divergent patterns of Rac1 activation following chronic stress are consistent with the loss and formation of dendritic spines in the hippocampus and amygdala respectively. But, the significance of the opposite effects of stress on Rac1 activation is not limited to morphological plasticity of spines, and may also have physiological consequences. For instance, the regulation of normal cytoskeletal stability is important for maintaining long-term changes in synaptic efficacy, such as long-term potentiation (LTP) and depression (LTD) (Koleske, 2013). Consistent with this, the same chronic stress paradigm used here has been shown to facilitate LTP in the lateral amygdala, while various forms of stress are known to impair hippocampal LTP, but enhance LTD, in the hippocampus (Kim and Diamond, 2002; Chattarji et al., 2015). Further, LTP is associated with enhanced F actin content in dendritic spines along with their enlargement (Fukazawa et al., 2003; Okamoto et al., 2004), whereas LTD is associated with spine shrinkage and an increase in G actin content (Okamoto et al., 2004). Interestingly, cofilin inactivation is also associated with spine stabilization and enlargement during LTP (Fukazawa et al., 2003; Ben Zablah et al., 2020). Thus, future studies will be necessary to further explore the physiological consequences of the divergent manifestations of stress-induced alterations in Rac1 signaling for synaptic transmission and plasticity in the hippocampus and amygdala.In conclusion, accumulating evidence has characterized how exposure to repeated stress triggers a range of structural and functional changes across biological scales—from behavior to synapses—that are strikingly different in the hippocampus vs. the amygdala. Our findings on the divergent effects of stress on Rac1-cofilin signaling in these two brain areas add a new dimension to this multi-level framework.

## Data Availability Statement

The original contributions presented in the study are included in the article/\hyperref[s9]**Supplementary Material**, further inquiries can be directed to the corresponding author.

## Ethics Statement

The animal study was reviewed and approved by The Institutional Animal Ethics Committee at the National Centre for Biological Sciences.

## Author Contributions

MB and SC designed the study. MB and MN conducted the experiments and analyzed data. MB, RP, and SC wrote the manuscript along with input from all the authors. All authors contributed to the article and approved the submitted version.

## Conflict of Interest

The authors declare that the research was conducted in the absence of any commercial or financial relationships that could be construed as a potential conflict of interest.

## Publisher’s Note

All claims expressed in this article are solely those of the authors and do not necessarily represent those of their affiliated organizations, or those of the publisher, the editors and the reviewers. Any product that may be evaluated in this article, or claim that may be made by its manufacturer, is not guaranteed or endorsed by the publisher.
