# Supplementary material for: Stress Elicits Contrasting Effects on Rac1-Cofilin Signaling in the Hippocampus and Amygdala
Source: Front Mol Neurosci. 2022 May 3;15:880382. doi: 10.3389/fnmol.2022.880382 (PMC9110925; doi:10.3389/fnmol.2022.880382)
Supplement: Supplementary file 1 [file Data_Sheet_1.PDF]

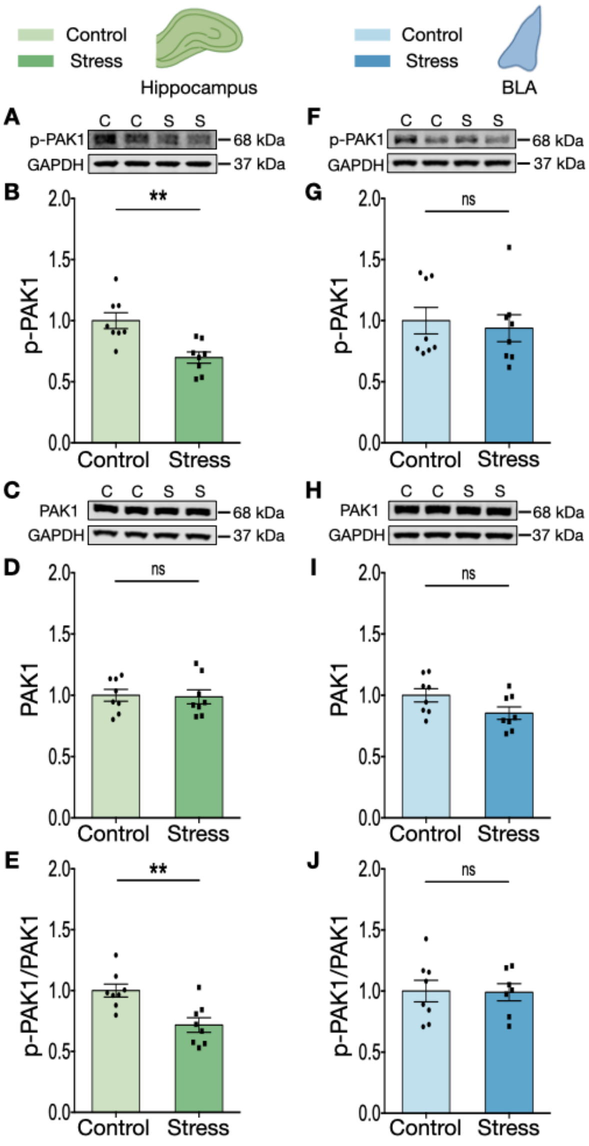

Supplementary Figure 1

Supplementary Figure 1: Activation profile and abundance of PAK1 protein in the whole tissue lysate isolated from the hippocampus and amygdala. (A-B) Significant decrease in the activity of PAK1 protein has been observed in the whole tissue lysate from the hippocampus of the stressed rats compared to the controls as shown in the representative western blot **(A)** and the summary graph **(B)** (Control: 1.00 ± 0.07; Stress: 0.70 ± 0.05; N = 8 rats/group; **p<0.01). **(C-D)** No change in the abundance of the PAK1 protein has been observed in the hippocampus of stressed rats as shown in the representative blot **(C)** and the summary graph **(D)** (Control: 1.00 ± 0.05; Stress: 0.99 ± 0.06; N = 8 rats/group). **(E)** Significant decrease in the ratio of phospho-PAK1 to total-PAK1 protein has been observed in the hippocampus due to stress (Control: 1.00 ± 0.05; Stress: 0.72 ± 0.06; N = 8 rats/group; **p<0.01). **(F-G)** No change in the activity of PAK1 protein has been observed in the amygdala of the stressed rats compared to the controls as shown in the representative western blot **(F)** and the summary data **(G)** (Control: 1.00 ± 0.11; Stress: 0.94 ± 0.11; N = 8 rats/group). **(H-I)** No change in the abundance of the PAK1 protein has been observed in the amygdala of stressed rats as shown in the representative blot **(H)** and the summary graph **(I)** (Control: 1.00 ± 0.05; Stress: 0.86 ± 0.05; N = 8 rats/group). **(J)** The ratio of phospho-PAK1 to total-PAK1 protein has been observed to remain unaltered in the amygdala due to chronic stress (Control: 1.00 ± 0.09; N = 8 rats; Stress: 0.99 ± 0.07; N = 7 rats).

In the figure, C stands for control, S stands for stress and ns stands for non-significant. Data are represented as means ± SEM and data normalized to control animals. The internal control used for analysis in Supplementary Figure 1B and Supplementary Figure 1G are same as in Supplementary Figure 2B and Supplementary Figure 2F respectively since they are from the same blots.

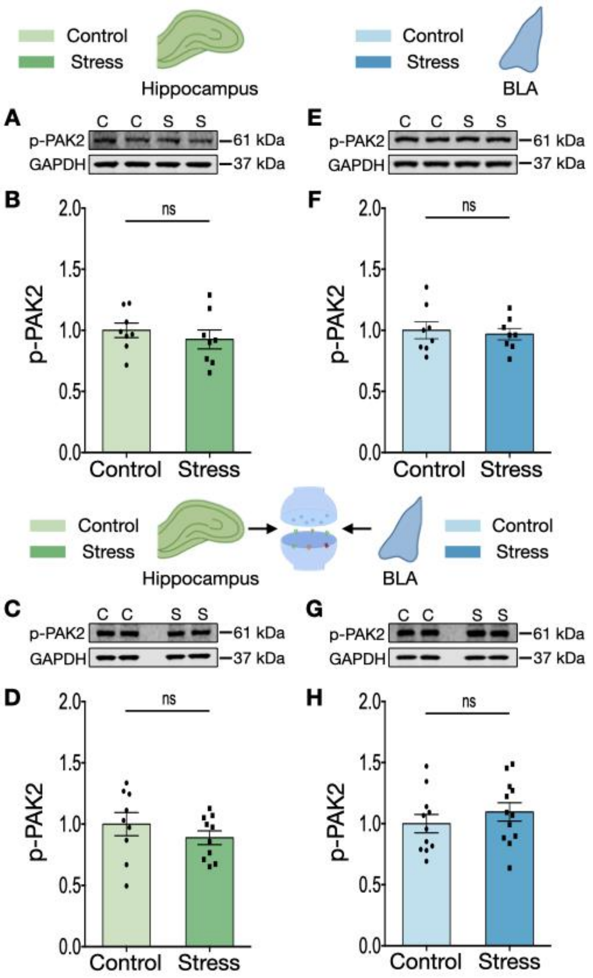

Supplementary Figure 2

Supplementary Figure 2: PAK2 activity remains unaltered after stress in the whole tissue lysate and synaptoneurosomes isolated from the hippocampus and the amygdala. (A-D) No change in the activity of the PAK2 isoform has been observed in the hippocampus due to stress as shown in the representative blots of tissue lysate **(A)** and synaptoneurosome fraction **(C)** as well as in the summary graphs **(B)** (Control: 1.00 ± 0.06; Stress: 0.93 ± 0.08; N = 8 rats/group) and **(D)** (Control: 1.00 ± 0.09; N = 9 rats; Stress: 0.89 ± 0.06; N = 10 rats) respectively. **(E-H)** The activity of the PAK2 isoform has been also observed to undergo no change in the amygdala due to stress as shown in the representative blots of tissue lysate **(E)** and synaptoneurosome **(G)** as well as the respective graphs **(F)** (Control: 1.00 ± 0.07; Stress: 0.97 ± 0.05; N = 8 rats/group) and **(H)** (Control: 1.00 ± 0.07; N = 11 rats; Stress: 1.10 ± 0.08; N = 12 rats).
In the figure, C stands for control, S stands for stress and ns stands for non-significant. Data are represented as means ± SEM and data normalized to control animals. The internal control used for analysis in Supplementary Figure 1B and Supplementary Figure 1G are same as in Supplementary Figure 2B and Supplementary Figure 2F respectively since they are from the same blots. Also, the same internal control has been used for analysis in Supplementary Figure 2D and Supplementary Figure 2H as in Figure 2B and Figure 2G respectively. The control GAPDH blot for Supplementary Figure 2A and Supplementary Figure 2E are same as Supplementary Figure 1A and Supplementary Figure 1F respectively and for Supplementary Figure 2C and Supplementary Figure 2G the control GAPDH blot is same as Figure 2A and Figure 2F respectively.
